# Numerical Investigation of T-Shaped Microfluidic Oscillator with Viscoelastic Fluid

**DOI:** 10.3390/mi12050477

**Published:** 2021-04-22

**Authors:** Chao Yuan, Hongna Zhang, Xiaobin Li, Masamichi Oishi, Marie Oshima, Qinghe Yao, Fengchen Li

**Affiliations:** 1 School of Aeronautics and Astronautics, Sun Yat-sen University, Guangzhou 510275, China; yuanch9@mail2.sysu.edu.cn; 2 School of Mechanical Engineering, Tianjin University, Tianjin 300350, China; lixiaobin@tju.edu.cn (X.L.); lifch@tju.edu.cn (F.L.); 3 Institute of Industrial Science, The University of Tokyo, Tokyo 153-8505, Japan; oishi@iis.u-tokyo.ac.jp (M.O.); marie@iis.u-tokyo.ac.jp (M.O.)

**Keywords:** microfluidic oscillator, oscillating flow, viscoelastic fluid, T-shaped channel, elastic instability

## Abstract

Oscillatory flow has many applications in micro-scaled devices. The methods of realizing microfluidic oscillators reported so far are typically based on the impinging-jet and Coanda effect, which usually require the flow Reynolds number to be at least at the order of unity. Another approach is to introduce elastomeric membrane into the microfluidic units; however, the manufacturing process is relatively complex, and the membrane will become soft after long-time operation, which leads to deviation from the design condition. From the perspective of the core requirement of a microfluidic circuit, i.e., nonlinearity, the oscillatory microfluidic flow can be realized via the nonlinear characteristics of viscoelastic fluid flow. In this paper, the flow characteristics of viscoelastic fluid (Boger-type) in a T-shaped channel and its modified structures are studied by two-dimensional direct numerical simulation (DNS). The main results obtained from the DNS study are as follows: (1) Both Weissenberg (Wi) number and viscosity ratio need to be within a certain range to achieve a periodic oscillating performance; (2) With the presence of the dynamic evolution of the pair of vortices in the upstream near the intersection, the oscillation intensity increases as the elasticity-dominated area in the junction enlarges; (3) Considering the simplicity of the T-type channel as a potential oscillator, the improved structure should have a groove carved toward the entrance near the upper wall. The maximum oscillation intensity measured by the standard deviation of flow rate at outlet is increased by 129% compared with that of the original standard T-shaped channel under the same condition. To sum up, with Wi number and viscosity ratio within a certain range, the regular periodic oscillation characteristics of Oldroyd-B type viscoelastic fluid flow in standard T-shaped and its modified channels can be obtained. This structure can serve as a passive microfluidic oscillator with great potential value at an extremely low Reynolds number, which has the advantages of simplicity, no moving parts and fan-out of two.

## 1. Introduction

A fluid oscillator is a device that utilizes the instability of fluid flow to produce a continuous pulsating jet. It has a wide variety of applications, such as jet mixing enhancement [1,2,3], heat transfer enhancement [4,5,6], cavity resonance suppression [7,8], etc. In addition, as a flow control actuator, the fluid oscillator is utilized in aerospace industries because of its unique features and its simplicity with no moving parts, such as missile control [9] and jet thrust vector control [10]. A comprehensive overview of the fluid oscillator can be found in the review literature [9,11].

The concept of the microfluidic oscillator has emerged since the size of the fluid oscillator device was reduced to microscale. There are many applications of microfluidic oscillator in microscale fields, such as particle sorting [12]/focusing [13], mixing/heat transfer enhancement at microscale [14,15], flowmeter [16,17,18], chemical process enhancement [19,20], filtration improvement [21] and hemodynamics [22]. Microfluidic oscillators can also be integrated with other fluidic components for more complex flow control and operation.

At present, the principle of using continuous fluid to realize the fluidic oscillator can be roughly divided into the following categories (as exemplified in detail by several typical literature articles in Table 1). (1) The equivalence principle of electronic and fluidic circuit. Lammerink et al. [23] designed several equivalent basic fluidic elements and assembled them into a multivibrator. (2) Coanda effect. A rapid jet enters the diffuser at a high aspect ratio and takes the nearby fluid element away from the wall. Therefore, the uneven low-pressure area is formed on both sides, and then the flow is randomly attached to one of the side walls. Xie et al. [24] and Yang et al. [25] studied the Coanda effect with symmetric and asymmetric feedback channels, respectively. (3) Impinging-jet-based principle. The flow instability can be induced by the jet impinging on a certain barrier [16,18] or by the two jets colliding at a certain angle [26,27], so as to realize the oscillating characteristics. The common features of the above design principles are that Newtonian fluid is used as the flow working fluid, and the flow Reynolds number (Re) ranges from about 10 to several thousands. However, as Re further decreases to an order of even smaller than unity, the inertial nonlinearity effect is usually so weak as to restrict the fluid flow to be laminar.

The core of a fluidic circuit is the nonlinearity [28]. As one of the components of a fluidic circuit at microscale, the microfluidic oscillator needs other non-inertial nonlinearity sources, which can be introduced in many ways. The methods of multiphase flow, such as micro bubbles [29,30]/droplets [31] and electrochemistry [32] for microfluidic control are complex in channel fabrication and lack stability or repeatability. Recently, a hydroelastic microfluidic oscillator [33,34], which uses an elastic diaphragm (silicone rubber) as the intermediate layer of the channel, was realized within a medium range of Re (~10–100). Similarly, a microfluidic oscillator was achieved from a channel with two valves made from an elastomeric membrane [35,36]. Nevertheless, the stiffness of the elastic diaphragm will decrease after a long period of operation, resulting in the overall reduction of the working pressure range of the hydroelastic microfluidic oscillator. In addition, the complexity of the channel increases the difficulty of its fabrication.

Despite the almost negligible inertial effects in a micro-scaled channel, the viscoelastic fluid can bring about complex flow phenomena, such as symmetry breaking, purely elastic instabilities [37,38] and even elastic turbulence [39,40], which arise from the combination of curved streamlines and large tensile stress. The anisotropic elastic stress arising from the natural nonlinear property of viscoelastic fluid flow itself is the source of strong nonlinearity, which meets the core requirements of fluidic circuit [28]. Taking the viscoelastic fluid as the working medium has the advantages of a single-phase medium and no moving parts compared with the abovementioned methods.

The application of oscillatory flow of viscoelastic fluid has been reported in several studies. Using the flow of viscoelastic fluid in the form of oscillation generated by external solenoid valve, Asghari et al. [41] achieved particle focusing and separation at nano-scale, which indicates that the same efficiency can be obtained by reducing the channel length from 4 cm to 4 mm. Sun et al. [42] studied the weakly shear-thinning fluid flowing in the microfluidic oscillator previously designed for Newtonian fluid. The results verified that the linear relationship between flow rate and frequency is still valid. All these studies involved viscoelastic fluid, but the typical properties of viscoelastic fluid have not been fully used to realize the oscillator.

The realization of a microfluidic oscillator via a simple structure is of great importance for eliminating the dependence on external instruments, integrating microfluidic components and reducing the failure rate of functional components. Therefore, the base geometry studied in this paper is a right-angled axisymmetric T-junction, which is a classical geometric model used in hemodynamics [43], droplet formation [44], oil transportation [45], etc. The purpose of choosing symmetrical geometry is to expect the average flow rate of both outlets to be similar, so that both exits can serve as the excitation source of pulsation. In fact, the study of unsteady flow through a T-junction is fairly common. Soulages et al. [46] investigated the T-shaped stagnation flow geometries with and without a short length of recirculating cavity, and found the onset of the symmetry-breaking transition with the presence of the cavity. Varshney et al. [47] experimentally studied a millimeter-sized T-type channel with a long recirculating cavity. They reported that in the range of similar Re and Wi numbers where forward bifurcation usually occurs, forward Hopf bifurcation was observed instead, and the existence of the long recirculating cavity played a decisive role. Poole et al. numerically investigated the effects of aspect ratio and shear-thinning properties for generalized Newtonian fluid [48] and viscoelasticity [49] on the symmetry-breaking bifurcations in T-channel flows. A common feature of the above studies is that two streams collided head-on from two opposite entrances and then evacuated through 90 degrees. In terms of the two possibilities of flow in or out of each port in the T channel, two streams can collide in perpendicular [44] or one stream enters from the left and leaves from both the exit in line with the inlet and the exit perpendicular to the inflow [43,50,51], as summarized in Table 2. So, naturally, we were very curious about the last case, that is, the fluid flowing out from two outlets in a straight line. Although seldom, studies like Colin et al.’s [45] have also adopted this kind of arrangement; they have focused on the impact of wormlike micelles on the T-junction flow, which is a classical model in the oil industry. The elastic instability of the viscoelastic fluid flow in the T channel with two streams leaving in opposite directions is still worthy of further study.

In this paper, two-dimensional direct numerical simulation (DNS) of viscoelastic fluid flow in a T-shaped channel as prototype and several modified structures is carried out to investigate the flow characteristics at a very low Re. The effect of viscosity ratio of the viscoelastic fluid is also studied. The remainder of this paper is arranged as follows. Section 2 introduces the numerical simulation methodology, including the description of governing equations, the verification of grid independence, etc. In Section 3, the results and discussions are presented mainly from the pulsing performance, the effect of Wi and viscosity ratio, and the stretching analysis of macromolecular structure. Finally, Section 4 ends with the conclusions, and the prospect of the future improvement of a T-shaped microfluidic oscillator is put forward.

## 2. Numerical Procedure

### 2.1. Physical Model

**Figure 1:**
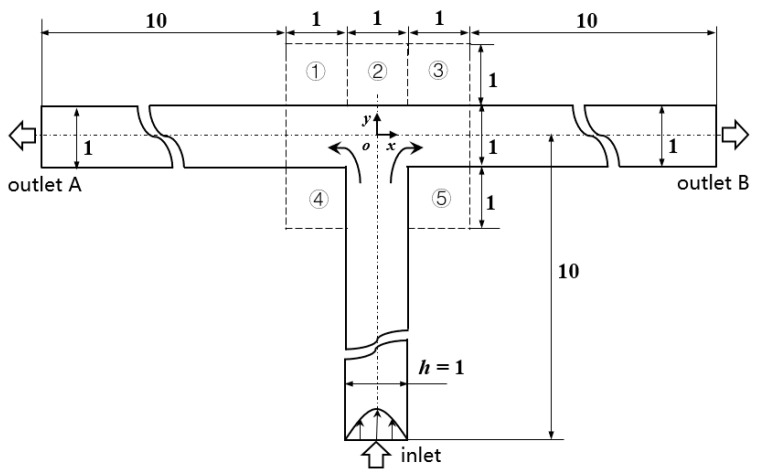
Schematic of the microfluidic oscillator channel.

Figure 1 shows the schematic of the flow unit studied in this paper. The basic structure is a T-shaped channel with one inlet and two outlets. Four modified structures were formed by adding cavities. The geometry description and symbol marking of the channels are summarized in Table 3.

### 2.2. Governing Equations and Numerical Method

Two-dimensional incompressible adiabatic flow was considered. The equations of conservations of mass and momentum were adopted. Generally, viscoelastic fluid has shear-thinning physical property, but to simplify the research complexity, Boger-type fluid with constant shear viscosity was selected in this paper, and the constitutive equation was Oldroyd-B [53]. This simplification is quite common for the convenience of research. [54,55]. For the sake of generality, each variable is nondimensionalized, and the dimensionless governing equations are the following.

(1) ∇·u=0,

(2) ∂u∂t+u·∇u=−∇p+βRe∇2u+1−βRe·Wi∇C,

(3) $$\frac{\partial C}{\partial t}+u\cdot \nabla C=C\cdot \nabla u+{\left(\nabla u\right)}^{T}\cdot C+\frac{1}{\mathrm{Wi}}\left(I-C\right)$$

where t is the time, u is the velocity, p is the pressure, ∇ is the nabla operator, I is the unit tensor and C is the conformation tensor, which is symmetric and positive definite. The Reynolds number is described as Re=(ρUh)/μ0, where ρ is the density, U is the average flow velocity in the inflow and h is the channel width. The Weissenberg number is defined as Wi=λU/h, where λ is the relaxation time of the fluid. The viscosity ratio is defined as $\beta =\mu_{s}/\left(\mu_{s}+\mu_{p}\right)=\mu_{s}/\mu_{0}$, where $\mu_{s}$ and $\mu_{p}$ are the dynamic viscosity of the solvent and the solute of viscoelastic fluid, respectively. $\mu_{0}$ is zero-shear rate viscosity. Unless otherwise specified, all data below are dimensionless.

As for the boundary conditions, the no-slip condition was imposed at walls, and the opening condition was applied at outlets. A fully developed velocity profile was set at the inlet to eliminate the influence of entrance. The elastic stress was relaxed at the initial moment, i.e., the conformation tensor was a unit tensor. The detailed description of numerical scheme and solution method are given in [56].

### 2.3. Grid Independence Validation

The standard T-shape geometry was used to verify the grid independence, and four sets of grids were adopted. Different numbers of uniform grids were arranged in the intersection center of the channel, and the same number of grid nodes were distributed in the three arms. Starting from the minimum mesh size, the mesh was distributed at a grid growth rate of 1.1 from the center to the outside. See Table 4 for the specific mesh settings. In this paper, the ranges of Wi and β were 0~10 and 0.1~0.9, respectively. An example with Wi=10 and β=0.1 was selected for grid independence verification. The time step for different mesh settings was 10^−4^ and Re was 0.01.

**Figure 2:**
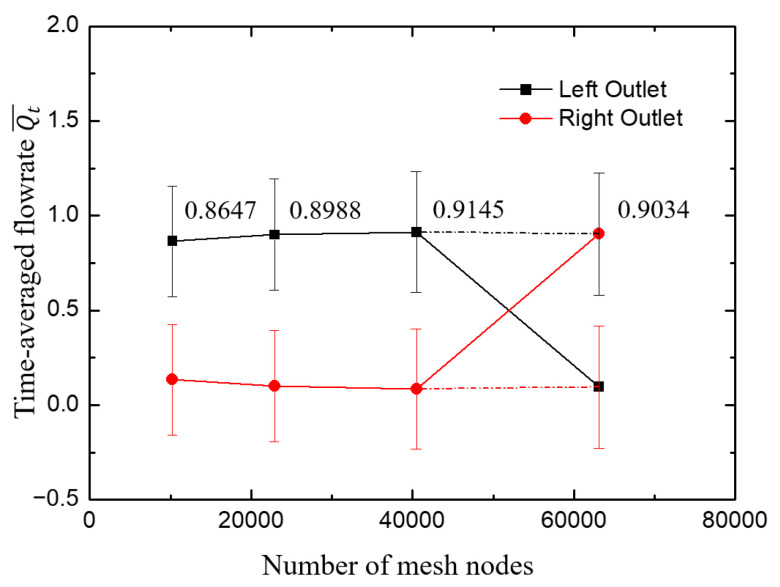
The change of time-averaged flow rate $\bar{Q_{t}}$ at outlets with grid number. The error bar is the standard deviation of instantaneous flow rate and the label denotes $\bar{Q_{t}}$ at the left outlet, with one exception of the last point corresponding to $\bar{Q_{t}}$ at the right outlet. The cross of the lines results from the random phenomenon in such flows as discussed above. To clarify this, the last two data are connected by dotted line.

**Figure 3:**
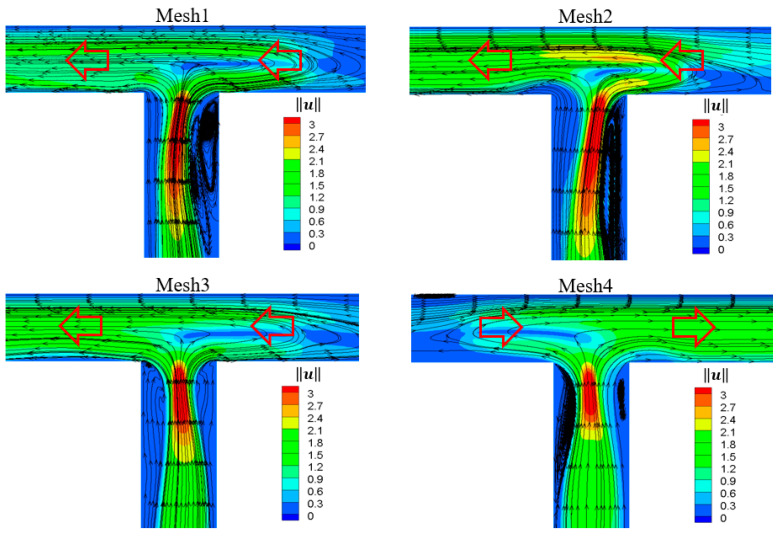
Contour plots of velocity magnitude ‖u‖ superimposed with streamlines for different sets of mesh. *t* = 114, Wi = 10, β=0.1, Re=0.01.

Figure 2 shows that the statistical time-averaged flow rates of the two outlets tended to be stable with the increase of grid number, and the difference between the results of Mesh3 and Mesh4 was 1.2%. Considering the computing capacity and accuracy, the node layout of Mesh3 was selected during the subsequent research. It is worth noting that the results of the two branch flows of Mesh4 were opposite to those of Mesh3. For the case of Mesh4, Figure 3 shows that the flow rate at the right outlet was so large that the fluid flow in the left arm reversed to the right side, which was contrary to the results of the other three sets of grids. This random phenomenon in the flow direction was reasonable and consistent with the experimental [57] and numerical simulation [58] results of viscoelastic fluid under the microfluidic cross-slot channel, indicating the randomness of viscoelastic fluid in elastic instability.

## 3. Results and Discussion

Considering the influence of the initial conditions, the result analyses were performed for only the last 700 out of the whole 1000 dimensionless simulated time units. The pulsing performance of the outlet flow was examined, which was mainly measured by the mean and variance of the average flow. Then, the stretching degree of microstructure (represented by the trace of conformation tensor) and the flow field were studied to analyze the phenomenon. The Re for all cases was fixed to 0.01 but with various Wi, indicating that the flow phenomenon appearing at such conditions could be only ascribed to the elastic effect of viscoelastic fluid.

### 3.1. Pulsing Performance of Viscoelastic Fluid Flow in Standard T-Shaped (ST) Channel

Variables oscillating with time are the typical features for an oscillator. Therefore, we first discuss the change of the pressure and velocity. To study the flow characteristics of viscoelastic fluid, three typical Wi of 1, 5 and 10 and three typical viscosity ratios *β* of 0.1, 0.5 and 0.9, respectively, were selected as the research objects. After analyzing the results, it was found that the flow under some working conditions had regular oscillation characteristics. This subsection first shows the results of the case at Wi = 5 and *β* = 0.5 with expected results in the ST channel.

**Figure 4:**
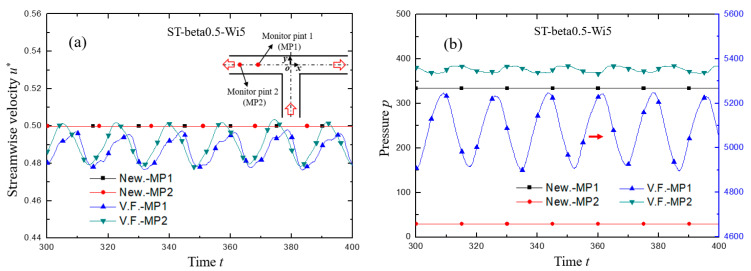
The variation of (**a**) streamwise velocity and (**b**) pressure with dimensionless time. In the legend, the symbols of MP1 and MP2 describe the monitor points of (*x*, *y*) = (−5, 0) and (*x*, *y*) = (−10, 0), respectively. The symbols of New. and V.F. denote respectively Newtonian and viscoelastic fluid. For both cases, Re = 0.01. For viscoelastic fluid, Wi = 5, *β* = 0.5.

**Figure 5:**
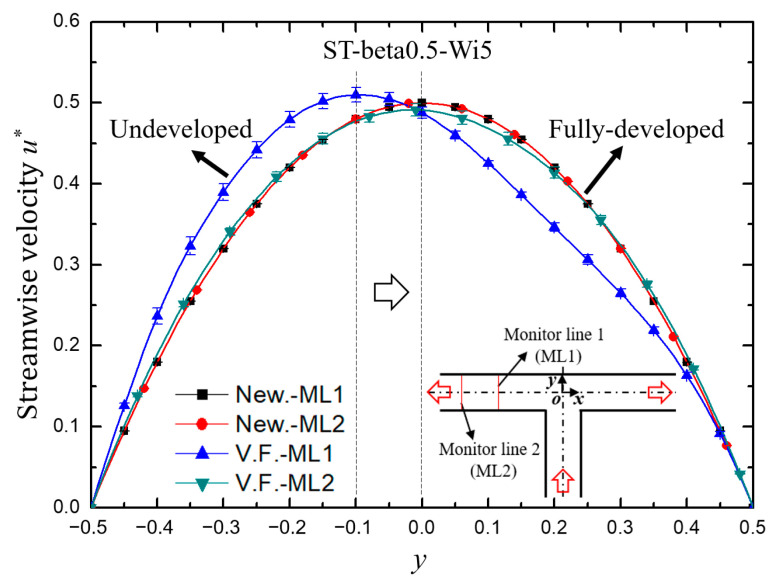
The velocity profile along the monitor lines of *x* = −5 (ML1) and *x* = −10 (ML2). The velocity u* is the time-averaged streamwise velocity of the last 700 dimensionless time units, and the error bar is the standard deviation.

The variation of streamline velocity u* normalized by the maximum velocity at inlet and pressure at monitored points of (*x*, *y*) = (−5, 0) and (*x*, *y*) = (−10, 0), respectively, are shown in Figure 4. It can be clearly seen that for Newtonian fluid, the velocity and pressure at the monitoring point did not change with time, because the Re was so small (Re = 0.01) that the flow was in laminar state. For viscoelastic fluid with enough elastic effect, however, the flow velocity and pressure showed regular periodic oscillation. In addition, the fluctuation form of velocity developed from irregular periodic arrangement at the upstream to nearly perfect sinusoidal-like waveform at the downstream, indicating that the flow gradually fell into a fully developed state. This was also concluded from the velocity profile across the channel as drawn in Figure 5. The velocity profile of Newtonian fluid in laminar flow is a standard parabolic shape, while the velocity distribution of viscoelastic fluid in the upstream undeveloped state is deviated from the standard parabolic shape, and the location of the maximum velocity is 10% of the channel width away from the center toward the lower wall. The fully developed velocity profile of viscoelastic fluid at the downstream is almost coincident with that of Newtonian fluid, but still inclines slightly to the lower wall.

**Figure 6:**
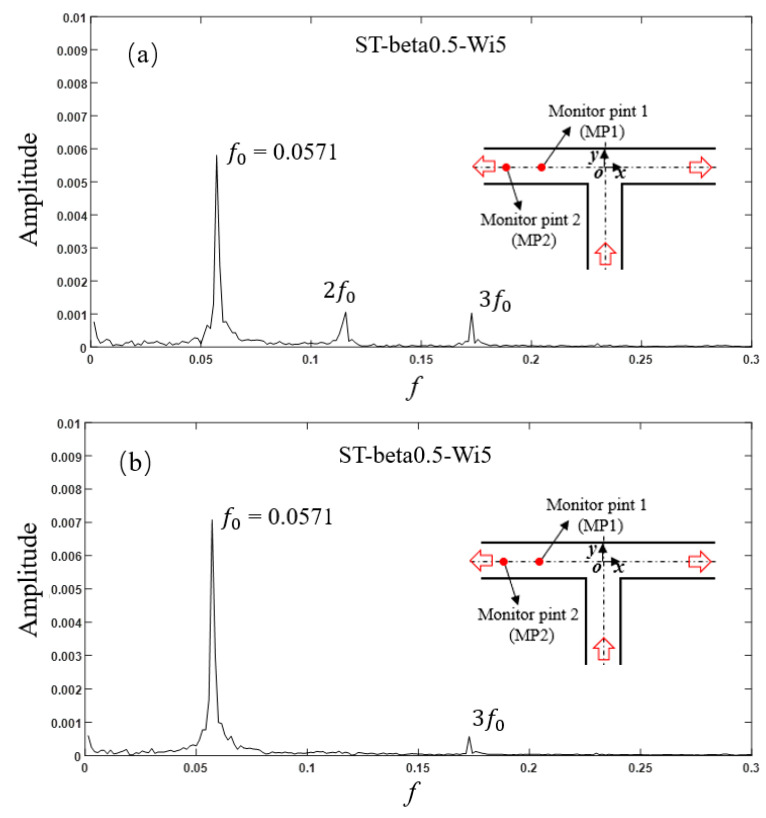
Amplitude spectrum of axial velocity fluctuation at (**a**) MP1 and (**b**) MP2 for the ST structure with *β* = 0.5, Wi = 5.

The amplitude spectrum of axial velocity fluctuation of viscoelastic fluid in the ST structure is shown in Figure 6. There existed a fundamental frequency ($f_{0}$ = 0.0571, the same value for the modified structures of CU1 and CU2) and two harmonic frequencies at (*x*, *y*) = (−5, 0). As the flow developed downstream, the amplitude of fundamental frequency increased, and that of the harmonic frequency weakened and some harmonic frequencies disappeared.

### 3.2. Effect of the Weissenberg Number and Viscosity Ratio

Given a constant average velocity unity at the inlet, the sum of the instantaneous average flow rate *Q_t_* of the two outlets should be unity, considering the conservation of continuity. For simplicity, only the result of the left branch of the channel is considered in the following discussion. When the flow pattern is completely symmetric, the *Q_t_* is equal to 0.5. Because of the possible complex flow, this parameter may exceed the range of 0 to 1, indicating that backflow may occur at an outlet.

**Figure 7:**
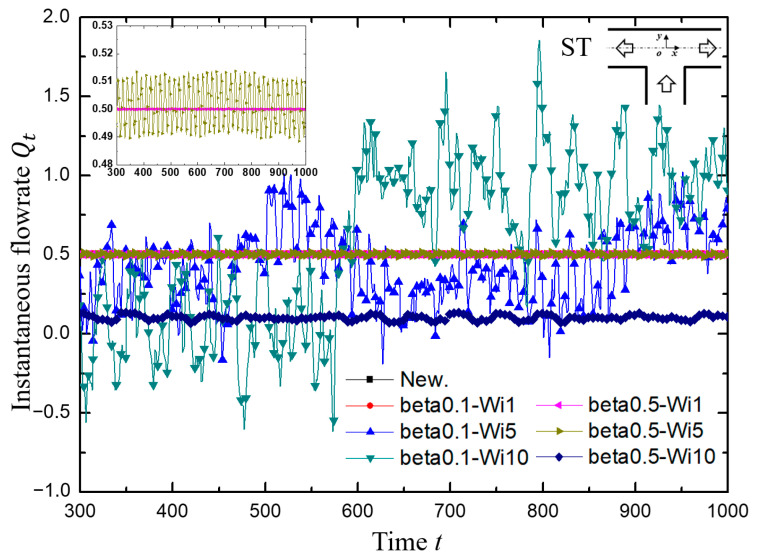
The variation of instantaneous flow rate *Q_t_* at the left outlet with dimensionless time. Inset: For the cases of Newtonian fluid, beta0.1-Wi1, beta0.5-Wi1 and beta0.5-Wi5, the *y*-axis ranges are set to 0.48 to 0.53 to display more clearly, where the former three cases are nearly collapsed into a line with a value of 0.5.

The variation of instantaneous flow rate with time is shown in Figure 7. The Weissenberg number Wi and viscosity ratio *β* measured the intensity of elastic effect, therefore the increasing of Wi and decreasing of *β* implied the greater roles of stretch-coil transition played in the viscoelastic fluid flow. (1) When Wi was low, such as in the cases of beta0.1-Wi1 in the red line and beta0.5-Wi1 in the magenta line, the curve of flow rate collapsed together into a straight line with *Q_t_* about 0.5, leading to the Newtonian-like fluid flow characteristics. (2) With Wi increased further, the elastic effect for the case of beta0.1-Wi5 (blue line) increased dramatically and the flow became randomly oscillating around the averaged flow rate with a high oscillation amplitude that was even beyond the normal range (0~1), indicating the appearance of backflow at one outlet. Interestingly, for the case of beta0.5-Wi5 in the dark yellow line, the change of instantaneous flow rate with time was approximately a periodic waveform with amplitude between 0.49 and 0.51, which provided a possibility to realize the viscoelastic fluid microfluidic oscillator having a periodic pulsing shape. (3) As Wi grew greater, in other words, the elastic effect playing a more important role in the fluid flow, the flow rate changed like a random process for the case of beta0.1-Wi10 in the green line. In sum, a desired oscillator can be obtained with viscoelastic fluid by carefully choosing the appropriate range of Wi.

### 3.3. Effect of the Channel Shape

**Figure 8:**
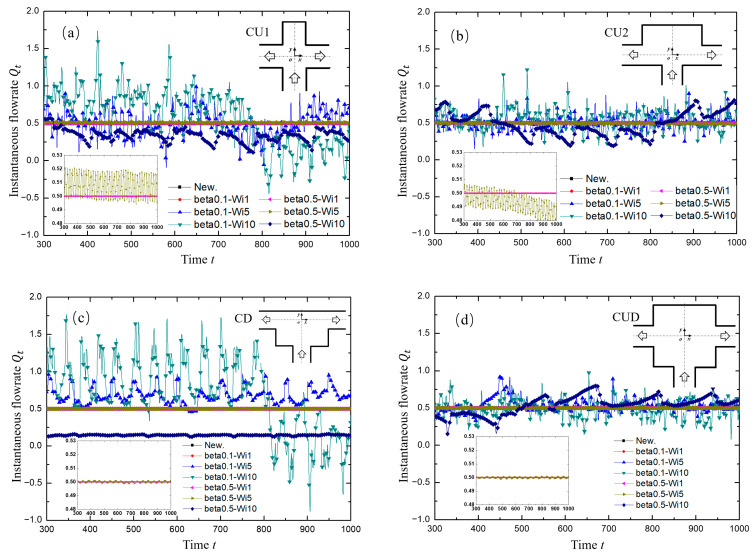
The variation of instantaneous flow rate *Q_t_* at the left outlet for different modified T-channel structures with dimensionless time. (**a**) CU1 structure; (**b**) CU2 structure; (**c**) CD structure; (**d**) CUD structure. Inset: For the cases of Newtonian fluid, beta0.1-Wi1, beta0.5-Wi1 and beta0.5-Wi5, the *y*-axis ranges are set to 0.48 to 0.53 to display more clearly, where the former three cases are nearly collapsed into a line with a value of 0.5.

**Figure 9:**
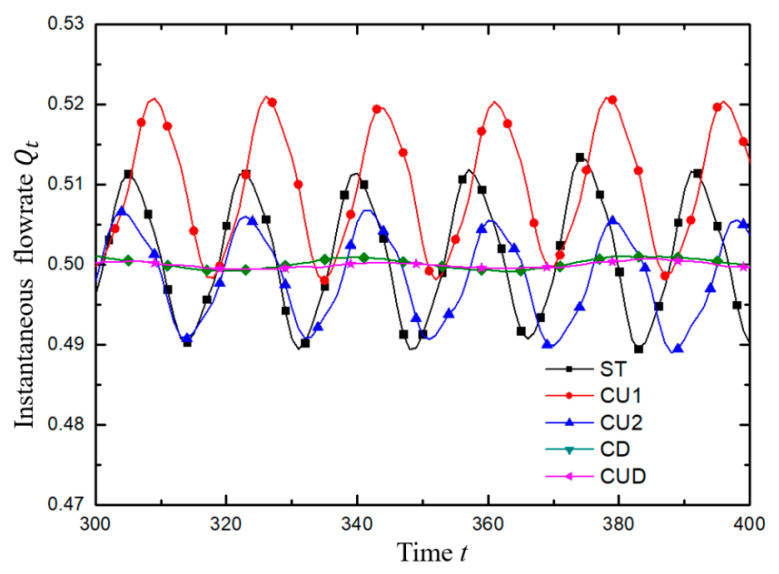
The variation of instantaneous flow rate *Q_t_* at the left outlet with dimensionless time. For all cases, β=0.5, Wi=5. The results in the dimensionless time range of 300 to 400 are an example to illustrate the approximate periodic waveform.

The variation of the instantaneous flow rate at the left exit of the four modified structures with various symmetrical square grooves around the intersection of the T-shaped channel is shown in Figure 8. The general results were similar to those of the standard T-shaped channel as discussed in Section 3.2. However, the cavities carved on different positions exerted an obviously unique impact on the flow field. In the case of beta0.1-Wi10 in the green line, for example, the wider upper cavities (CU2 and CUD) seemed to have a suppression influence on the pulsing flow in terms of the amplitude. In addition, the results of beta0.5-Wi5, an example of medium viscosity with medium Wi, deserve special attention. Firstly, as shown in the subgraphs in Figure 8 and more clearly demonstrated in Figure 9, for the standard T-type and the two cases of CU1 and CU2, the instantaneous average flow rate at the outlet presented approximately periodic fluctuation. However, for the two cases of CD and CUD with grooves at two lower corners, the instantaneous average flow rate at the outlet presented a weak fluctuation with a very small amplitude compared with the first three cases, as displayed in Table 5. Secondly, the closer the average value of the outlet flow rate approached to 0.5, the more likely the exits on both sides could be used as the pulsation excitation sources. In other words, the fan-out of the device could become two. In brief, considering both the pulsation intensity and the fan-out, the ST structure was the most suitable for the viscoelastic fluid microfluidic oscillator when β = 0.5 and Wi = 5 in this study. In addition, when the viscosity ratio was medium but the Wi was high, as shown with diamond symbols in Figure 8a, for example, the flow presented a certain regular pulsation, but the flow was easy to flip with different flow rates at the two outlets, as shown with diamond symbols in Figure 8b, for example, which was not easy to control.

**Figure 10:**
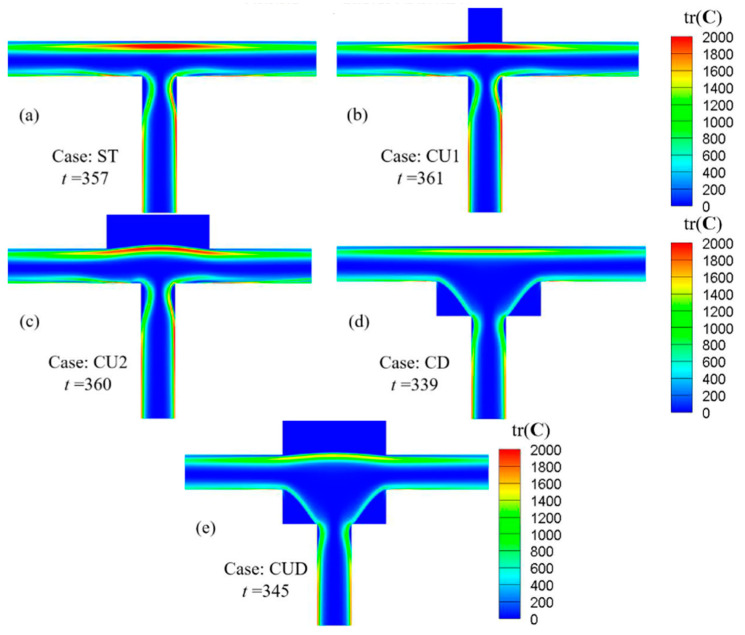
Distribution of the trace of conformation tensor tr(**C**) for various geometries at the instant with the largest flow rate in Figure 9. For all cases, β=0.5, Wi=5.

To understand the motion of viscoelastic fluid under the conditions of β=0.5 and Wi=5, the variation of microstructure stretching in viscoelastic fluid flow was analyzed, which was tracked by the trace of conformation tensor tr(**C**) [59]. Figure 10 shows the distributions of tr(**C**) for various geometries at the instant with the largest flow rate in Figure 9. In the ST channel, the relatively large tr(**C**) region dominated by shear stress tended to be close to the vertical axis of symmetry at the throat in the inflow channel, while after the flow entered the junction, the macromolecular structure was stretched greatly at the stagnation point dominated by tensile stress, and a severe stretching zone was formed near the upper wall. The situations of cases CU1 and CU2 were similar to that of ST. However, for the geometries with square recesses added to the lower walls (CD and CUD), the relatively large tr(**C**) region tended to be far away from the vertical axis of symmetry at the throat due to the smaller pairs of vortices, as shown in Figure 12. In addition, compared with the structures without cavities at the two lower corners (ST, CU1 and CU2), the range of low tr(**C**) region increased in the junction due to the presence of sudden expansion structure.

**Figure 11:**
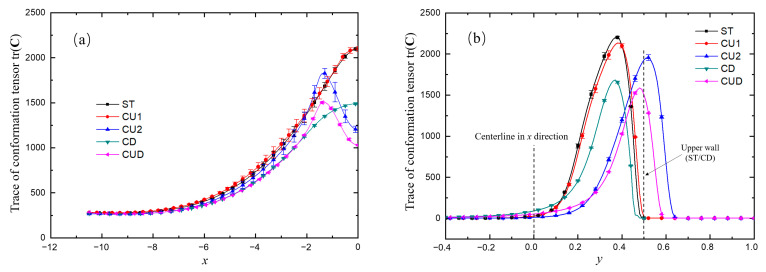
Variation trends of the time-averaged trace of conformation tensor tr(**C**) along the lines of (**a**) *y* = 0.4 and (**b**) *x* = 0. For all cases, β=0.5, Wi=5.

Figure 11 shows the variation trends of time-averaged tr(**C**) along the two axes. It can be seen from Figure 10 that the position where the maximum tension occurred in the horizontal channel was close to the upper wall, which had an important influence on the results. Therefore, Figure 11a shows the variation trend of tr(**C**) on the horizontal line with *y* = 0.4. All the curves are symmetrical about the vertical axis, and only the left halves are plotted. The stagnation points of ST and CD structures are fixed on the intersection of the horizontal wall and the vertical axis, and their tr(**C**) takes the maximum values locally. However, for the structures of CU2 and CUD with grooves on the upper side, their stagnation points are free to move into the groove, as shown in Figure 10c,e. Therefore, the maximum values of tr(**C**) are located in the groove, and the corresponding curves on the axis of symmetry in Figure 11a take the local minimum value. It is worth noting that the width of the groove also had an important effect on the flow. The results of the CU1 structure with smaller groove width were similar to those of the ST structure, which seemingly indicated that the smaller groove size had almost no effect on the flow. The variation of tr(**C**) on the centerline of axis *y* shown in Figure 11b shows again that the maximum tensile position of the structure with a wider groove on the upper side is inclined to the side of the upper groove.

### 3.4. Mechanism Analysis

**Figure 12:**
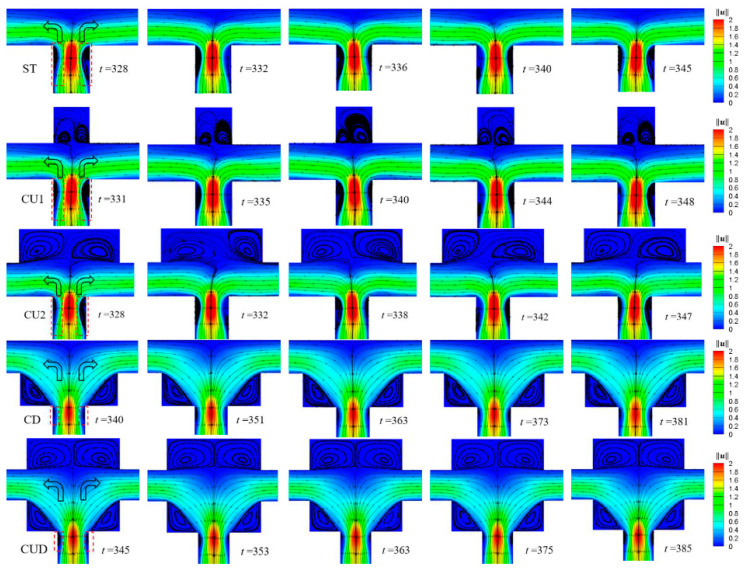
Contour plots of velocity magnitude ‖u‖ superimposed with streamlines for cases of ST, CU1, CU2 CD and CUD from the top to the bottom. The time of the five columns of graphs approximately corresponds to (n+0)T, (n+1/4)T, (n+1/2)T,(n+3/4)T and (n+1)T of the period of the regular wave, where *T* is the period corresponding to the fundamental frequency of each case. β=0.5, Wi=5. Note that the first and third columns denote the instants when the mass flow rate of left channel is the maximum and minimum in a period, respectively.

As described above, the viscoelastic flows in the ST channel and channels with symmetrical modifications on the upper wall (CU1 and CU2) exhibited approximately sinusoidal-like oscillation behaviors at the two outlets. The local flow conditions near the junction were responsible for the oscillatory flow downstream the exit channels. As depicted in Figure 12, for the geometries without cavities at the lower corners (ST, CU1 and CU2), a pair of lip vortices formed in the entrance branch at the throat region moved up and down periodically just as the periodically changing flow rate at outlets shown in Figure 9. For the cases of CD and CUD, only a pair of small vortices were generated in the throat region, which were not strong enough to impart significant effect on the flow field in the downstream, resulting in a relatively steady flow both in the mainstream and in the cavities. Also, it can be seen from Figure 12 that the velocity magnitude at the lower part of the horizontal branches was higher than that at the upper part, which directly explained the fact that the undeveloped velocity profile as shown in Figure 5 bent to the lower wall.

**Figure 13:**
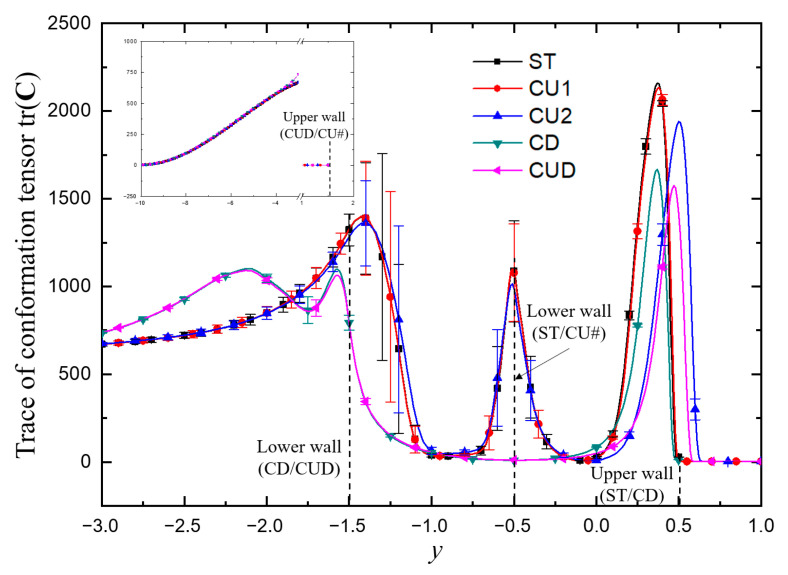
The time-averaged trace of conformation tensor tr(**C**) changes along the line of *x* = −0.4 showing the local near-wall information about molecular structure stretching in the throat region of the T-shaped channels. The error bar is the standard deviation of tr(**C**). Inset: the range of y covers from *y* = −10 (the inlet) to *y* = −3 and from *y* = 1 to *y* = 1.5, where no peaks or valleys of tr(**C**) profiles appear. For all cases, β=0.5, Wi=5.

**Figure 14:**
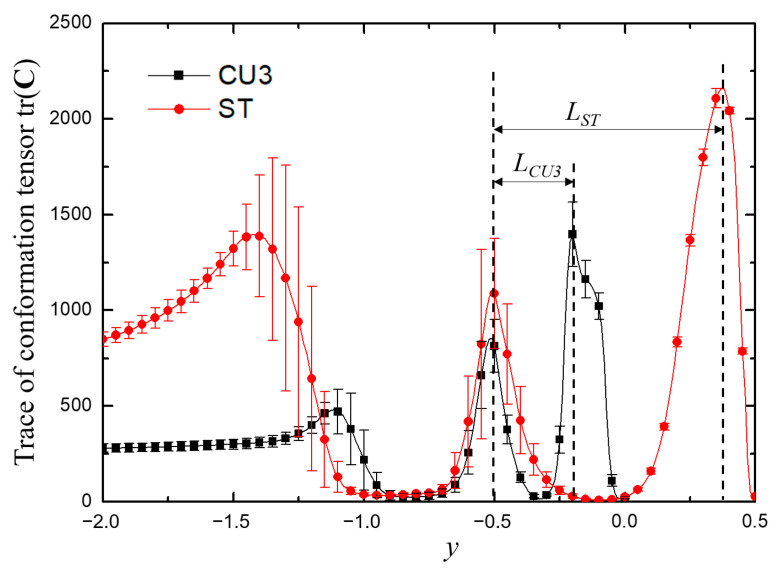
The time-averaged trace of conformation tensor tr(**C**) changes at the line of *x* = − 0.4. For all cases, β=0.5, Wi=5. *L_ST_* and *L_CU3_* are the distance between the second and third maxima of tr(**C**), which is a measure of the elastic effect in the junction domain for the cases of ST and CU3, respectively.

The viscoelastic fluid in the entrance branch was only subjected to strong shear action near the wall. As illustrated previously, the dynamics of the vortex pair in the throat region played an important role. Therefore, we took the variation of tr(**C**) along the line *x* = −0.4 (close to the wall) to analyze the microstructure stretching quantitatively as displayed in Figure 13. The stretching of microstructures induced the peculiar flow behaviors. It can be seen from Figure 13 that, along this line close to the wall, the stretching extent of the molecular structure increased and decreased three times alternately. The first minimum was related to the vortex at the upstream of the sudden expansion, while the second was due to the weak shear effect of the main flow near the center of the horizontal channel, and the last was related to the low-speed motion near the upper wall (ST and CD) or the grooves on the upper side (CU1, CU2 and CUD). Here, we used *L* (the distance between the second and third maxima of tr(**C**), as shown in Figure 14) to measure the elastic effect in the junction domain. The smaller *L* showed a stronger elastic effect in the whole junction domain. As shown in Figure 13 and Figure 14, it indicated that a T-shaped-based structure with a smaller *L* in the junction tends to be more efficient as a microfluidic oscillator.

**Figure 15:**
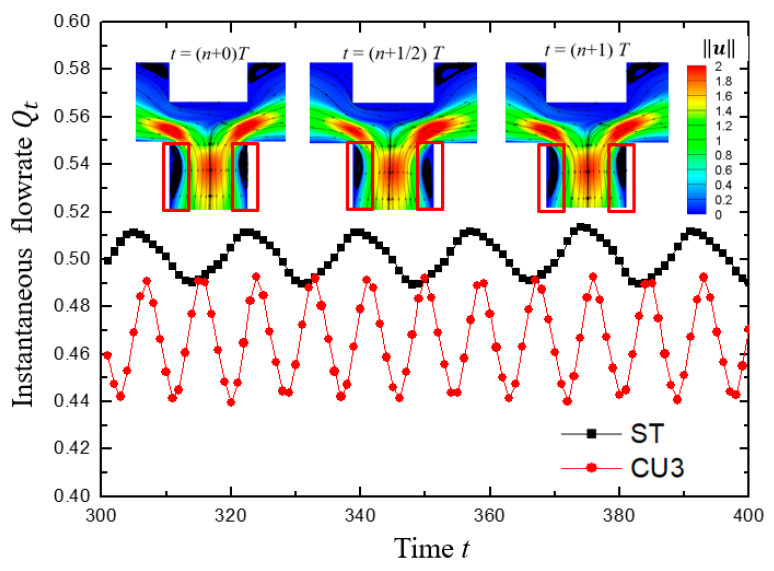
The variation of instantaneous flow rate *Q_t_* at the left outlet with dimensionless time for the case of standard T-shaped channel (ST) and modified channel with cavity on the top wall with half-channel width carved out (CU3). For all cases, β=0.5, Wi=5. Inset: Three instants for contour plots of velocity magnitude ‖u‖ superimposed with streamlines. *T* is the period corresponding to the fundamental frequency of the CU3 structure.

To further verify that the oscillation characteristics were related to the periodic fluctuation of the upstream vortex pair and the distance *L*, viscoelastic fluid flow in a channel with a groove carved inward on the upper wall (denoted as case CU3, inspired by the reference [60]) was also simulated under the same condition with β=0.5 and Wi=5, as shown in Figure 15 for typical results. Half-channel width was chosen as the characteristic length for CU3. The results showed that the standard deviation of the outlet flow rate $\sigma \left(Q_{t}\right)$ was increased by 129% (calculated by $\left(\sigma {\left(Q_{t}\right)}_{\mathrm{CU}3}-\sigma {\left(Q_{t}\right)}_{\mathrm{ST}}\right)/\sigma {\left(Q_{t}\right)}_{\mathrm{ST}}\times 100\%$, where $\sigma {\left(Q_{t}\right)}_{\mathrm{CU}3}$ and $\sigma {\left(Q_{t}\right)}_{\mathrm{ST}}$ are the standard deviation of $Q_{t}$ for cases of CU3 and ST, respectively) from 0.0075 to 0.0172, compared with the case ST. Note that the averaged flow rate $\bar{Q_{t}}$ at the left outlet was 0.4661, deviated from the ideal value of 0.5. As drawn in Figure 14, the distance *L* of case CU3 is dramatically decreased compared to that of the case ST. It is worth noticing that the oscillation frequency of the case CU3 increased sharply from 0.0571 of the case ST to 0.1250, implying that the oscillating performance is closely related to the channel geometry in addition to the physical properties of viscoelastic fluid.

The results of viscosity ratio *β* = 0.9 were similar to those of Newtonian fluid, therefore the corresponding results are not presented in this paper. In sum, the pulsation intensity of the ST and its modified geometry in this study were at most only 3.7% of the mean value. For now, regarding the practical application of the so-called microfluidic oscillator as an “excitation source” in microfluidic circuits, there seems to be a certain distance between the research and reality. The geometry form of CU3 that had an obvious improved performance was not optimized systematically, but was a preliminary structure obtained by mechanism analysis. Inspired by the elegant and ingenious structure proposed by predecessors (for example, a variant of switch structure in [28]), we can expect to get a better performance of the simple T-shaped microfluidic oscillator with viscoelastic fluid by carefully designing the core components, together with three-dimensional simulation and later experimental verification.

## 4. Conclusions and Outlook

In this paper, the flow of Boger-type viscoelastic fluid in two-dimensional standard T-shaped and its modified structures was numerically simulated. The main conclusions are drawn as follows:

1. For viscoelastic fluid with medium viscosity ratio and medium Wi the average flow rate at the outlets of standard T-shaped and its modified structures changes approximately sinusoidal-like with time;
2. To generate regular periodic signals, both Wi and viscosity ratio need to be within a certain range, beyond which no oscillation or completely irregular flow will occur;
3. The mechanism of the oscillation characteristics is related to the periodic fluctuation of the upstream vortex pair and the size of the elasticity-dominated area in the whole junction domain. With the presence of the dynamic evolution of the pair of vortices in the upstream near the intersection, the oscillating intensity increases as the elasticity-dominated area in the junction enlarges;
4. When the T-type simple structure is considered as the potential realization of the oscillator, for now, the modified structure with a groove carved inwards on the upper wall facing the entrance branch is the most suitable for the oscillator to provide excitation for the downstream equipment.

At present, the results obtained above are based on the constitutive equation of the Oldroyd-B model, which is suitable for the case where the stretching length of molecules is much less than the maximum extensibility. The extent of microstructure stretching (measured by the trace of conformation tensor tr(**C**)) for the considered ranges of Wi and β in various geometries in this paper reached values in the order of 2000, as displayed in the Figure 10. Given the more accurate simulation when the stretching length tends to the maximum stretching length under consideration, the application of the FENE model to carry out related research work will also be necessary in the future. Moreover, only sinusoidal-like periodic pulsating flow with limited amplitude is generated with two-dimensional simulation. To make full use of the T-type channel, which has the advantages of simplicity, no moving parts and fan-out of two, a lot of work still needs to be done, including ingenious design of the channel shape and careful selection of the appropriate flow condition range. As a next step of research, a thorough three-dimensional simulation using a realistic fluid model with finite extensibility such as the FENE model and meticulous microchannel experiments using real viscoelastic fluids are required to realize a T-type microfluidic oscillator with considerable pulsation amplitude to excite the downstream flows.

## Figures and Tables

**Table 1:** Various fluidic oscillators based on different principles.

Category	Characteristic	Channel Shape	Channel Width (μm)	Channel Depth (μm)	Fluid/Model	Re	*f* (Hz)	Reference
Electronic–fluidic analogy based	Fluidic circuit is analogous to electronic astable multivibrator	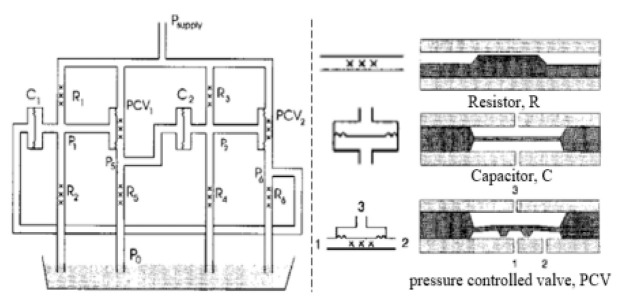	~30	n/a	Water; CAMAS program package	n/a	0.18	[23] ^a^
Coanda effect	Symmetric feedback channel	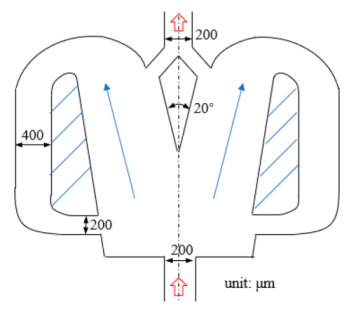	200	n/a	DI water; DNS	16.7~100	~180	[24] ^a^
	Asymmetric feedback channel	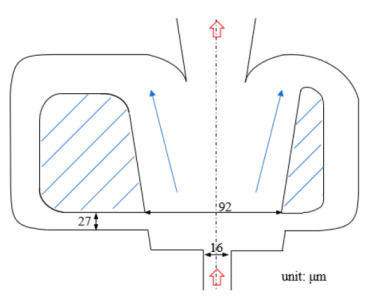	16	263	DI water; DNS	1~100	~1	[25] ^a^
Elastic diaphragm	The hydroelasticity realized by a four-layer substrate with a diaphragm embedded in between introduces nonlinearity	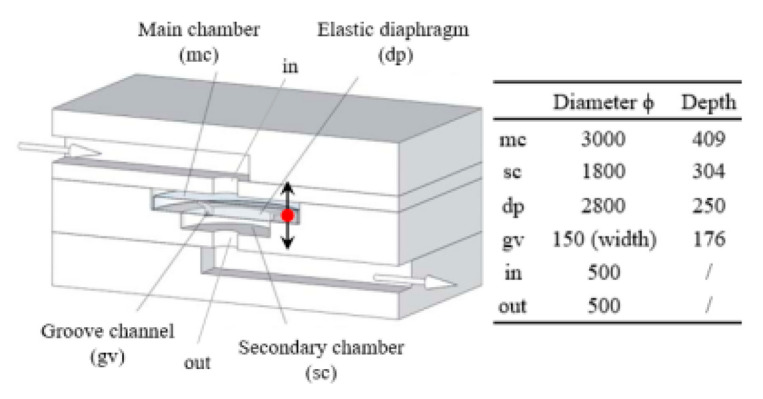	2800	n/a	50% glycerol–50% water solution	10~100	~210	[33] ^b^
	The combination of switch-valves and check-valves that both contain a diaphragm as a core element	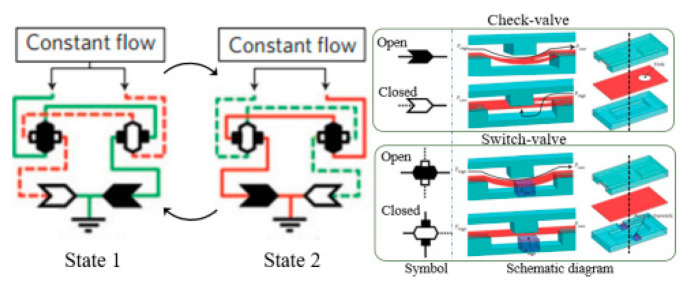	n/a	100	Water LabVIEW	n/a	~1	[52] ^a^
Impinging-jet-based	A planar jet impinges on a V-shaped plate downstream	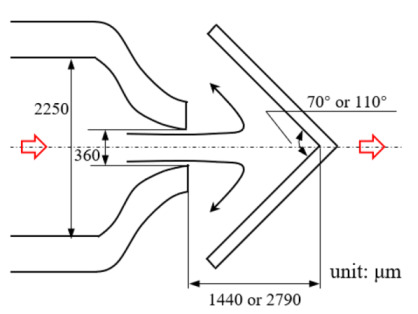	1440, 2790	48	water	0.2~5.4	~0.3	[16] ^b^
	Secondary flow induces Gortler instability	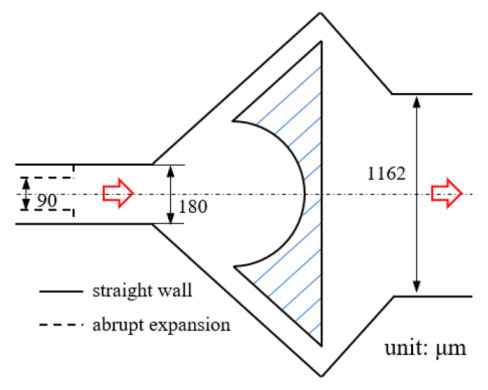	180	218.1	distilled water	50~450	0.1~0.6	[18] ^b^
	Interaction of two jets with intersecting angles	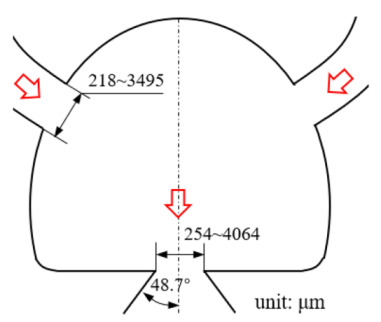	254~4064	152.4~2423	air, argon, hydrogen, pure water and sodium iodide solution; SST model	~10^4^	~3 × 10^4^ (hydrogen)	[26] ^a^
	Interaction between two opposing jets	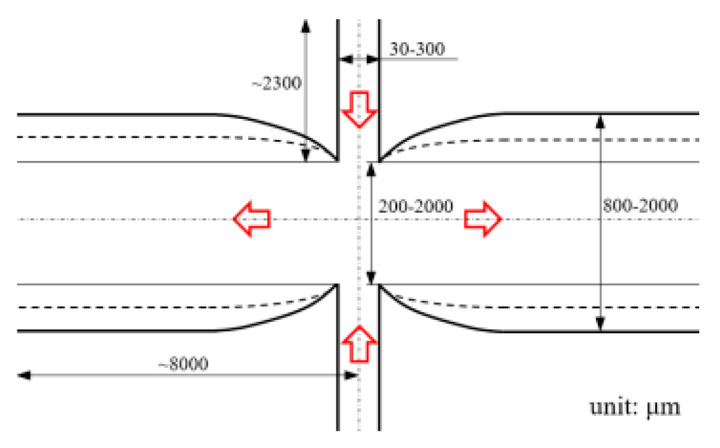	50~300	150~525	DI water; DNS	15~630	1230	[27] ^a^

Note: DI water, Deionized water. ^a^: Results obtained by both simulation and experiment; ^b^: Results obtained only by experiment. n/a: Not applicable.

**Table 2:** A summary of different flow patterns of viscoelastic fluids in various typical T-shaped structures in recent years.

Category	Exp/Num ^a^	Channel Shape	Width *h* (μm)	Fluid/Model	Wi	β	Re	Reference
Two streams collide head-on	Exp	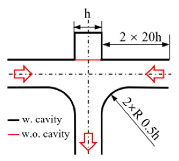	50	0.075 wt.% PEO (MW: 2 × 10^6^)	29.33	0.5	0.065	[46]
				glycerol/water mixture (60/40 wt.%)	n/a	n/a		
	2D		n/a	sPTT	4.4	0.5	~0	
	Exp	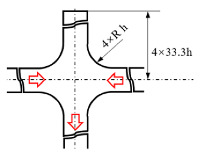	3000	80 ppm PAAm (MW: 18 × 10^6^)	~1200	~0.77	0.1~3	[47]
Two streams leave in perpendicular	2D	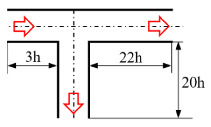	n/a	FENE-CR and FENE-MCR	3	0.3–0.9	102	[43]
	3D	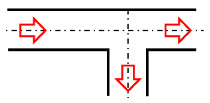	3100	Oldroyd-B	1.2 ^b^	0.9	50	[50]
	2D		n/a	Carreau–Yasuda	50~1000	n/a	n/a	[51]
Two streams collide in perpendicular	Exp	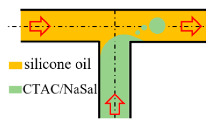	100	200, 600 and 1000 ppm CTAC with NaSal	1.54~42.22	n/a	n/a	[44]
Two streams leave in opposite directions	3D	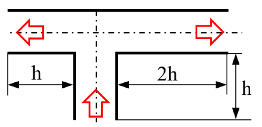	1000	modified Giesekus	n/a	n/a	~0	[45]

Note: PEO: polyethylene oxide; CTAC: cetyltrimethylammonium chloride; NaSal: sodium salicylate; PAAm: polyacrylamide; MW: Molecular weight. ^a^ The abbreviations for Exp and Num denote the results of experiments and simulations, respectively. Simulation includes two types: two-dimensional (2D) and three-dimensional (3D). ^b^ The data is estimated from the raw data in the original paper.

**Table 3:** Description of different channel structures and numerical database.

Case	Channel Geometry ^a^	Channel Shape	Case Illustration ^b^	*β*	Wi	Re
Standard T (abbreviated as “ST”)	n/a	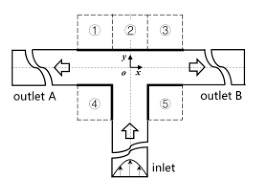	ST-N	1	0	0.01
			ST-betaXX-WiXX	0.1, 0.5, 0.9	1, 5, 10	
Cavity_Up1 (abbreviated as “CU1”)	②	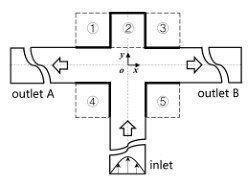	CU1-N	1	0	
			CU1-betaXX-WiXX	0.1, 0.5, 0.9	1, 5, 10	
Cavity_Up2 (abbreviated as “CU2”)	①②③	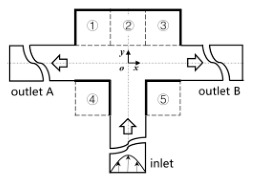	CU2-N	1	0	
			CU2-betaXX- WiXX	0.1, 0.5, 0.9	1, 5, 10	
Cavity_Down (abbreviated as “CD”)	④⑤	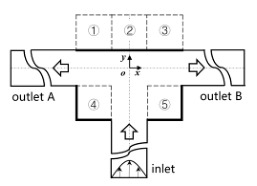	CD-N	1	0	
			CD-betaXX- WiXX	0.1, 0.5, 0.9	1, 5, 10	
Cavity_Up_Down (abbreviated as “CUD”)	①②③④⑤	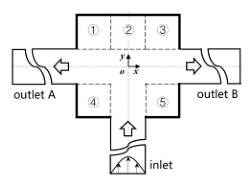	CUD-N	1	0	
			CUD-betaXX-WiXX	0.1, 0.5, 0.9	1, 5, 10	

^a^: The number is in accordance of that in Figure 1. ^b^: ST-N indicates that the geometric structure is a standard T-junction and the working medium is Newtonian fluid. ST-beta0.1-Wi1 is an example indicating a viscosity ratio of 0.1 and a Wi of 1. Other cases are similarly indicated.

**Table 4:** Characteristics of the meshes used in the grid independence validation.

Item	ND*_x_*	ND*_y_*	ND_branch_	$\Delta x_{min}$ and $\Delta y_{min}$	NC	ND
Mesh1	51	51	51	0.020	10,508	10,251
Mesh2	76	76	76	0.013	23,258	22,876
Mesh3	101	101	101	0.010	41,008	40,501
Mesh4	126	126	126	0.008	63,758	63,126

Note: ND*_x_* and ND*_y_* are the number of nodes in the *x* and *y* directions, respectively. ND_branch_ is the number of nodes along the three branches. $\Delta x_{min}$ or $\Delta y_{min}$ is the minimum mesh size in the *x* or *y* direction. NC and ND are the total number of cells and nodes, respectively.

**Table 5:** Statistical results over the last 700 dimensionless time. $\bar{Q_{t}}$ and $\sigma \left(Q_{t}\right)$ are the mean and standard deviation of $Q_{t}$, respectively.

	ST	CU1	CU2	CD	CUD
$\bar{Q_{t}}$	0.5015	0.5077	0.4935	0.5000	0.4999
$\sigma \left(Q_{t}\right)$	0.0075	0.0076	0.0066	0.0006	0.0005

## Nomenclature

C	conformation tensor
Wi	Weissenberg number
*f*	frequency
*f*_0_	fundamental frequency
*h*	channel width
I	unit tensor
L	A measure of the elastic effect area in the junction domain
p	pressure
*Q_t_*	instantaneous flow rate
$\bar{Q_{t}}$	mean flow rate
Re	Reynolds number
*t*	time
*T*	period
u	velocity vector
*u**	normalized streamwise velocity
U	average flow velocity in the inflow
x, y	coordinates
Greek symbols	
β	ratio of solvent dynamic viscosity to zero-shear viscosity
λ	relaxation time of viscoelastic fluid
σ	standard deviation
$\mu_{0}$	zero-shear fluid viscosity
$\mu_{p}$	dynamic viscosity of the solute
$\mu_{s}$	dynamic viscosity of the solvent
ρ	density
$\tau^{p}$	elastic stress
Superscripts	
T	transpose operator
Subscripts	
*i*, *j*	tensor representation

## References

[1] Tesar V. (2009). Oscillator micromixer. Chem. Eng. J..

[2] Dennai B., Khelfaoui R., Benyoucef B., Abdenbi A. (2012). Flow control mono and bi-stable fluidic device for micromixer-injection system. Energy Procedia.

[3] Dennai B., Bentaleb A., Chekifi T., Khelfaoui R., Abdenbi A. (2015). Micro fluidic oscillator: A technical solution for micro mixture. Adv. Mat. Res..

[4] Narumanchi S., Kelly K., Mihalic M., Gopalan S., Hester R., Vlahinos A. Single-phase self-oscillating jets for enhanced heat transfer. Proceedings of the Semitherm Conference.

[5] Tesar V. (2009). Enhancing impinging jet heat or mass transfer by fluidically generated flow pulsation. Chem. Eng. Res. Des..

[6] Lundgreen R.K., Hossain M.A., Prenter R., Bons J.P., Gregory J., Ameri A. Impingement Heat Transfer Characteristics of a Sweeping Jet. Proceedings of the 55th AIAA Aerospace Sciences Meeting.

[7] Raman G., Cain A.B. (2002). Innovative actuators for active flow and noise control. J. Aerosp. Eng..

[8] Raman G., Raghu S. (2004). Cavity Resonance Suppression Using Miniature Fluidic Oscillators. AIAA J..

[9] Gregory J., Tomac M.N. A Review of Fluidic Oscillator Development. Proceedings of the 43rd Fluid Dynamics Conference.

[10] Raman G., Packiarajan S., Papadopoulos G., Weissman C., Raghu S. (2005). Jet thrust vectoring using a miniature fluidic oscillator. Aeronaut. J..

[11] Khalde C.M., Pandit A.V., Sangwai J.S., Ranade V.V. (2019). Flow, mixing, and heat transfer in fluidic oscillators. Can. J. Chem. Eng..

[12] Dietsche C., Mutlu B.R., Edd J.F., Koumoutsakos P., Toner M. (2019). Dynamic particle ordering in oscillatory inertial microfluidics. Microfluid. Nanofluid..

[13] Mutlu B.R., Edd J.F., Toner M. (2018). Oscillatory inertial focusing in infinite microchannels. Proc. Natl. Acad. Sci. USA.

[14] Benavides E.M. (2009). Heat transfer enhancement by using pulsating flows. J. Appl. Phys..

[15] Wu J.W., Xia H.M., Zhang Y.Y., Zhao S.F., Zhu P., Wang Z.P. (2019). An efficient micromixer combining oscillatory flow and divergent circular chambers. Microsyst. Technol..

[16] Lee G.B., Kuo T.Y., Wu W.Y. (2002). A novel micromachined flow sensor using periodic flapping motion of a planar jet impinging on a V-shaped plate. Exp. Therm. Fluid Sci..

[17] Gregory J.W., Sullivan J.P., Raghu S. (2007). Characterization of the microfluidic oscillator. AIAA J..

[18] Sun C.-L., Sun C.-Y. (2011). Effective mixing in a microfluidic oscillator using an impinging jet on a concave surface. Microsyst. Technol..

[19] Zheng M., Mackley M. (2008). The axial dispersion performance of an oscillatory flow meso-reactor with relevance to continuous flow operation. Chem. Eng. Sci..

[20] Phan A.N., Harvey A., Lavender J. (2011). Characterisation of fluid mixing in novel designs of mesoscale oscillatory baffled reactors operating at low flow rates (0.3–0.6 mL/min). Chem. Eng. Process..

[21] Olayiwola B., Walzel P. (2009). Effects of in-phase oscillation of retentate and filtrate in crossflow filtration at low Reynolds number. J. Membr. Sci..

[22] Li Z., Dey P., Kim S.-J. (2019). Microfluidic single valve oscillator for blood plasma filtration. Sens. Actuators B Chem..

[23] Lammerink T.S.J., Tas N.R., Berenschot J.W., Elwenspoek M.C., Fluitman J.H.J. Micromachined hydraulic astable multivibrator. Proceedings of the IEEE Micro Electro Mechanical Systems.

[24] Xie T.L., Xu C. (2017). Numerical and experimental investigations of chaotic mixing behavior in an oscillating feedback micromixer. Chem. Eng. Sci..

[25] Yang J.T., Chen C.K., Hu I.C., Lyu P.C. (2007). Design of a Self-Flapping Microfluidic Oscillator and Diagnosis with Fluorescence Methods. J. Microelectromech. Syst..

[26] Tomac M.N., Gregory J. Frequency Studies and Scaling Effects of Jet Interaction in a Feedback-Free Fluidic Oscillator. Proceedings of the 50th AIAA Aerospace Sciences Meeting including the New Horizons Forum and Aerospace Exposition.

[27] Bertsch A., Bongarzone A., Duchamp M., Renaud P., Gallaire F. (2020). Feedback-free microfluidic oscillator with impinging jets. Phys. Rev. Fluids.

[28] Groisman A., Enzelberger M., Quake S.R. (2003). Microfluidic memory and control devices. Science.

[29] Prakash M., Gershenfeld N. (2007). Microfluidic bubble logic. Science.

[30] Stucki J.D., Guenat O.T. (2015). A microfluidic bubble trap and oscillator. Lab Chip.

[31] Toepke M.W., Abhyankar V.V., Beebe D.J. (2007). Microfluidic logic gates and timers. Lab Chip.

[32] Zhan W., Crooks R.M. (2003). Microelectrochemical logic circuits. J. Am. Chem. Soc..

[33] Xia H.M., Wang Z.P., Fan W., Wijaya A., Wang W., Wang Z.F. (2012). Converting steady laminar flow to oscillatory flow through a hydroelasticity approach at microscales. Lab Chip.

[34] Xia H.M., Wu J., Wang Z. (2017). The negative-differential-resistance (NDR) mechanism of a hydroelastic microfluidic oscillator. J. Micromech. Microeng..

[35] Kim S.J., Yokokawa R., Lesher-Perez S.C., Takayama S. (2015). Multiple independent autonomous hydraulic oscillators driven by a common gravity head. Nat. Commun..

[36] Kim S.-J., Yokokawa R., Lesher-Perez S.C., Takayama S. (2011). Constant Flow-Driven Microfluidic Oscillator for Different Duty Cycles. Anal. Chem..

[37] McKinley G.H., Pakdel P., Oztekin A. (1996). Rheological and geometric scaling of purely elastic flow instabilities. J. Non-Newton. Fluid..

[38] Pakdel P., McKinley G.H. (1996). Elastic instability and curved streamlines. Phys. Rev. Lett..

[39] Groisman A., Steinberg V. (2000). Elastic turbulence in a polymer solution flow. Nature.

[40] Yuan C., Zhang H.N., Li Y.K., Li X.B., Wu J., Li F.C. (2020). Nonlinear effects of viscoelastic fluid flows and applications in microfluidics: A review. Proc. Inst. Mech. Eng. Part C J. Eng. Mech. Eng. Sci..

[41] Asghari M., Cao X., Mateescu B., van Leeuwen D., Aslan M.K., Stavrakis S., deMello A.J. (2020). Oscillatory Viscoelastic Microfluidics for Efficient Focusing and Separation of Nanoscale Species. ACS Nano.

[42] Sun C.-l., Lin Y.J., Rau C.-I., Chiu S.-Y. (2017). Flow characterization and mixing performance of weakly-shear-thinning fluid flows in a microfluidic oscillator. J. Non-Newton. Fluid.

[43] Matos H.M., Oliveira P.J. (2014). Steady flows of constant-viscosity viscoelastic fluids in a planar T-junction. J. Non-Newton. Fluid.

[44] Li X.-B., Li F.-C., Kinoshita H., Oishi M., Oshima M. (2014). Dynamics of viscoelastic fluid droplet under very low interfacial tension in a serpentine T-junction microchannel. Microfluid. Nanofluid..

[45] Colin M., Colin T., Dambrine J. (2016). Numerical simulations of wormlike micelles flows in micro-fluidic T-shaped junctions. Math. Comput. Simulat..

[46] Soulages J., Oliveira M.S.N., Sousa P.C., Alves M.A., McKinley G.H. (2009). Investigating the stability of viscoelastic stagnation flows in T-shaped microchannels. J. Non-Newton. Fluid.

[47] Varshney A., Afik E., Kaplan Y., Steinberg V. (2016). Oscillatory elastic instabilities in an extensional viscoelastic flow. Soft Matter.

[48] Poole R.J., Alfateh M., Gauntlett A.P. (2013). Bifurcation in a T-channel junction: Effects of aspect ratio and shear-thinning. Chem. Eng. Sci..

[49] Poole R.J., Haward S.J., Alves M.A. (2014). Symmetry-breaking bifurcations in T-channel flows: Effects of fluid viscoelasticity. Procedia Eng..

[50] Keslerova R., Trdlicka D. (2015). Numerical solution of viscous and viscoelastic fluids flow through the branching channel by finite volume scheme. J. Phys. Conf. Ser..

[51] Miranda A.I.P., Oliveira P.J., Pinho F.T. (2008). Steady and unsteady laminar flows of Newtonian and generalized Newtonian fluids in a planar T-junction. Int. J. Numer. Methods Fluids.

[52] Mosadegh B., Kuo C.H., Tung Y.C., Torisawa Y.S., Bersano-Begey T., Tavana H., Takayama S. (2010). Integrated elastomeric components for autonomous regulation of sequential and oscillatory flow switching in microfluidic devices. Nat. Phys..

[53] Bird R.B., Curtiss C.F., Armstrong R.C., Hassager O. (1987). Dynamics of Polymeric Liquids: Kinetic Theory.

[54] van Buel R., Stark H. (2020). Active open-loop control of elastic turbulence. Sci. Rep. UK.

[55] Canossi D.O., Mompean G., Berti S. (2020). Elastic turbulence in two-dimensional cross-slot viscoelastic flows. EPL.

[56] Yuan C., Zhang H.N., Chen L.X., Zhao J.L., Li X.B., Li F.C. (2020). Numerical Study on the Characteristics of Boger Type Viscoelastic Fluid Flow in a Micro Cross-Slot under Sinusoidal Stimulation. Entropy.

[57] Sousa P.C., Pinho F.T., Oliveira M.S.N., Alves M.A. (2015). Purely elastic flow instabilities in microscale cross-slot devices. Soft Matter.

[58] Davoodi M., Domingues A., Poole R. (2019). Control of a purely elastic symmetry-breaking flow instability in cross-slot geometries. J. Fluid Mech..

[59] Zhang H.N., Li D.Y., Li X.B., Cai W.H., Li F.C. (2017). Numerical simulation of heat transfer process of viscoelastic fluid flow at high Weissenberg number by log-conformation reformulation. J. Fluid. Eng. T. ASME.

[60] Yeh L.H., Hsu J.P. (2009). Electrophoresis of a finite rod along the axis of a long cylindrical microchannel filled with Carreau fluids. Microfluid. Nanofluid..

